# Supplementary material for: Multi-neuron intracellular recording in vivo via interacting autopatching robots
Source: eLife. 2018 Jan 3;7:e24656. doi: 10.7554/eLife.24656 (PMC5812718; doi:10.7554/eLife.24656)
Supplement: Supplementary file 3. — The Supplementary file 3 describes all the materials and procedures to assemble the signal and pressure control hardware necessary to operate the multipatcher. [file elife-24656-supp3.docx]

**Supplementary File 3.**

**Assembly of the Control Box.**

The multipatcher control hardware was assembled by adapting the design used for the single channel autopatcher. We initially designed the multipatcher control hardware to be able to control up to 6 independent patch pipettes and ended up using 4 channels in the final version – the channel count being limited by the size of the robotic manipulator arms. Many steps for assembling the hardware are identical to the assembly of the autopatcher control box, and the assembly manual we published previously (Kodandaramaiah et al. 2016) has been adapted here.

**Tools Needed**:

1. Antistatic mat, grounded. (e.g., Digikey SCP225-ND.)‬

2. Antistatic wrist strap, grounded. (e.g., Digikey SCP172-ND.)‬

3. Soldering iron & solder. (e.g., Mouser 578-WES51.)

4. “Helping hands” for clamping components to be soldered. (e.g., Sparkfun TOL-11784.)

5. Wire cutters (“snips”) for cutting wire and cable ties. (e.g., Digikey 170M-ND.)

6. Hex key (a.k.a. Allen wrench), for ¼”-20 hex-head screws. (e.g., McMaster Carr 5709A31.)

7. Torx screwdrivers, sizes T25 and T30. (e.g., McMaster Carr 5756A45.)

8. Flathead screwdrivers, assorted sizes. (e.g., McMaster Carr 7020A51.)

9. Wire strippers. (e.g., Digikey JIC-2030-ND.)

10. 2x small, adjustable wrenches. (e.g., McMaster Carr 5385A11 and 5385A12.)

11. Multimeter. (e.g., Digikey 705-1051-ND.)‬

12. Handheld manometer for testing finished box. (e.g., Dwyer 475-5-FM.) ‬

13. Scissors for cutting Teflon tape and 1/8” o.d. tubing.

14. Razor knife for cutting ¼” and larger o.d. tubing.

15. Tape measure.

**Preassembly Preparation**

The overall schematic of the autopatcher control box is shown in **Fig. AM1**


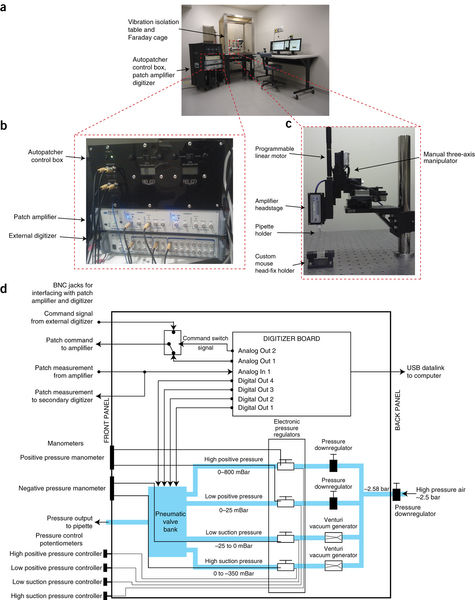


**Figure AM1:** Schematic of the single channel autopatcher robot (Kodandaramaiah et al 2016). The multipatcher robot uses the same architecture for pressure control signal switching in each channel (**Fig. 1, Supplements 1 and 2** in the main manuscript).

As illustrated here, the increased number of channels required a pneumatic valve bank that could independently deliver the discrete pressure states to each of the pipettes. We also used a data acquisition system (Compact DAQ, National Instruments Inc.) that allowed us to have 6 Analog Input channels, 7 Analog Output channels, and 20 digital TTL control signals. Secondly, the hardware for the single channel was designed to fit into one 9” high 19” rack mount box. As it was impossible to fit all the hardware in one unit, we split the pressure regulation hardware and the signal switching hardware into two 9” high 19” wide rack mount boxes (**Fig. 1, Supplements 1 and 2; Fig. AM2**).


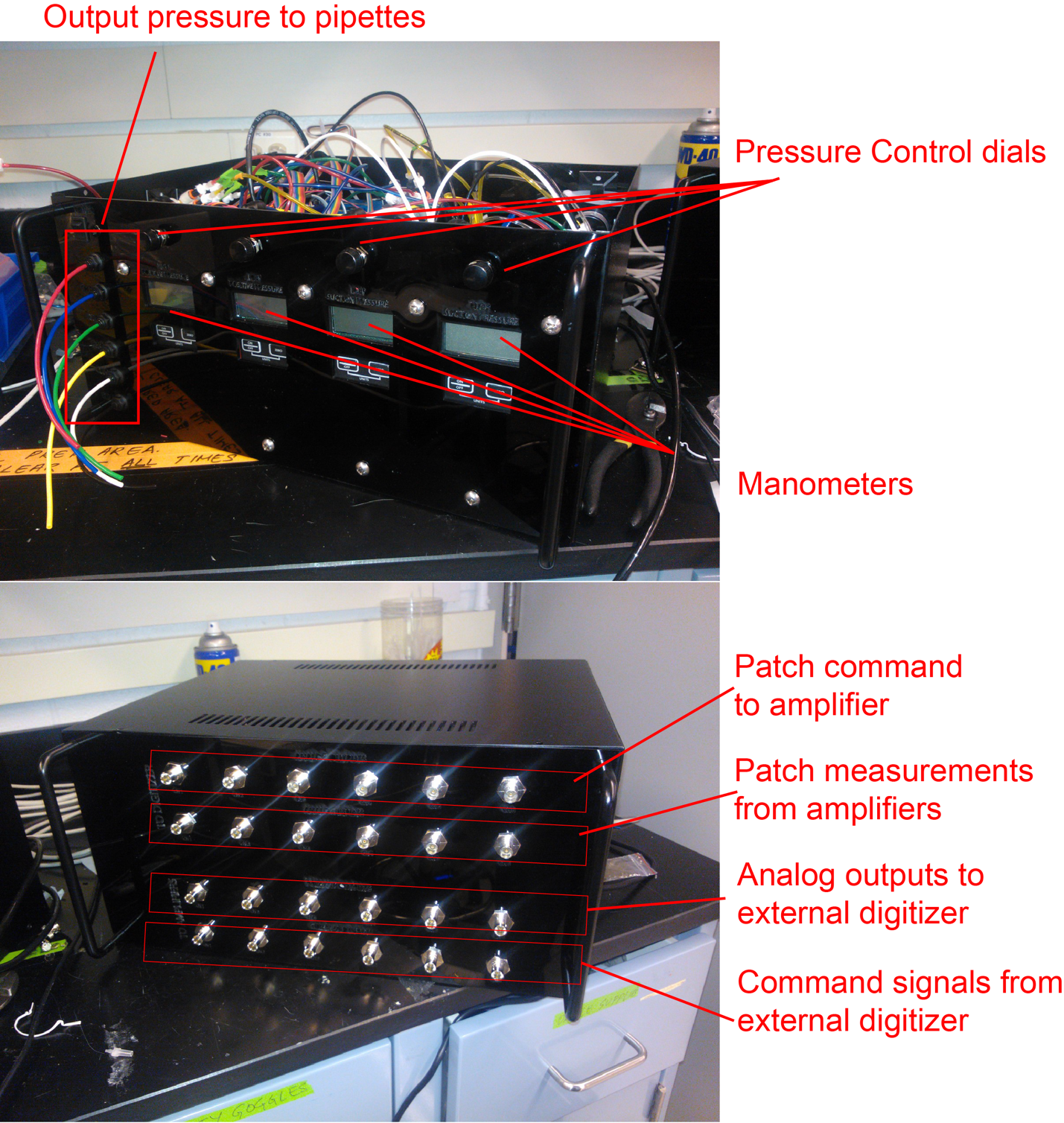


**Fig. AM2:** Photographs of the multipatcher pressure control box (top) and signal control box (bottom)

The Signal Control Box and the Pressure Control Box can be assembled following the instructions below.

**STEP 1.** Order the parts in the parts list file “Multipatcher parts list.xlsx”.


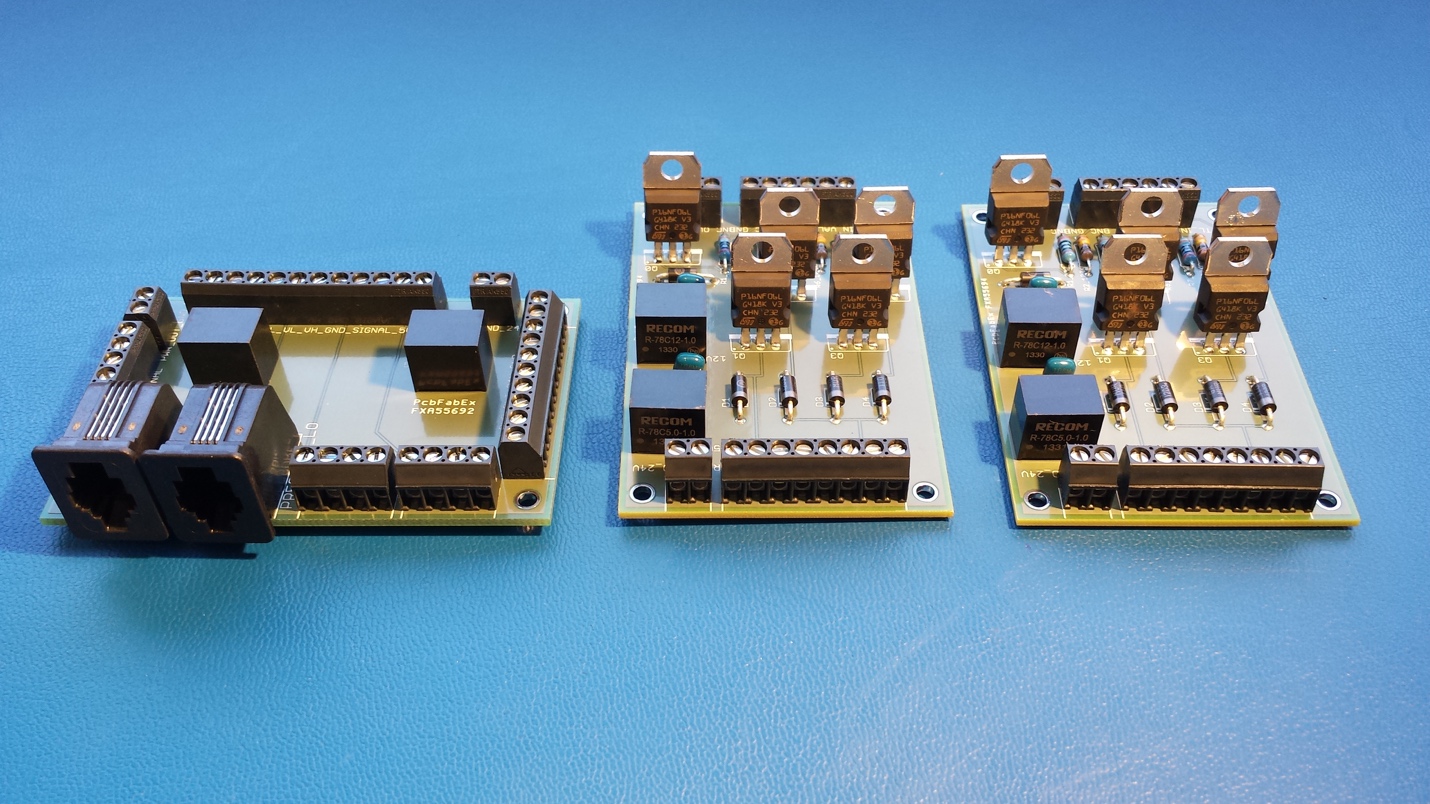


**Figure AM3:** The pressure regulator board is shown in the left on the left and two valve switching boards are shown on the right. You will need a total of n+1 valve switching boards for a n channel multipatcher control box. Reproduced, with permission, from Figure S3.1 of Kodandaramaiah et al. Nature Protocols 11, 634–654 (2016).

The assembled printed circuit boards can be seen in **Figure AM3**. The Gerber files included within the ‘Hardware assembly supplementary file.zip’ compressed file archive can be sent to a PCB fabrication house (e.g., pcbfabexpress.com) for procuring the prefabricated boards. These boards and the required components can then be sent to an assembly house (e.g., pcacorporation.com) for assembly or assembled in house. Two lists of the components used to populate the boards are included with the Gerber files and are called “pressure_board_2012-10-11_04_bom.xlsx” and “valve-relay_board_2012-11-16__04_bom.xlsx”. In general, if the multipatcher robot has *n* channels, you will need one pressure board and *n* + 1 valve boards. The pressure regulator board acts as an interface between the NI-Data acquisition board and electronic pressure regulators. The pressure switching valve takes regulated pressure outputs from the electronic pressure regulators and delivery a single pressure state to the pipette. One board controlling four solenoid valves is required for each pipette. Additionally, one board is used for electronic switching between manually regulated and programmatically controlled pressure.

**STEP 2:** Use the provided laser cutter files to cut the front and rear panels for the two control boxes along with the structural panels. The structural panels can be made out of ¼ acrylic, and the front and back panels can be cut out of 1/16” acrylic. Files named ‘Multipatcher_signalswitch_frontandbackpanel.ai’, ‘Multipatcher_structural base platform & manometer clamp.ai’, and ‘Mpatcher_fluidicsbox_frontandbackpanel.ai’ have been included in the supplementary materials. This step can be outsourced to a fabrication shop as well (e.g., customlasercutting.com).

**Figure AM4:** Rendering of front and back panels of the multipatcher signal control box. Use ‘Multipatcher_signalswitch_frontandbackpanel.ai’ to cut in a laser cutter. Black indicates cut lines, blue indicates etch lines and red are reference lines to outline the components that are mounted on the plate.

**Figure AM5:** Rendering of front and back panels of the multipatcher pressure control box. Use ‘Mpatcher_fluidicsbox_frontandbackpanel.ai’ to cut in a laser cutter. Black indicates cut lines, blue indicates etch lines and red are reference lines to outline the components that are mounted on the plate.

**Figure AM6:** Rendering of structural panels used in the multipatcher control box units. Black indicates cut lines, blue indicates etch lines and red are reference lines to outline the components that are mounted on the plate.


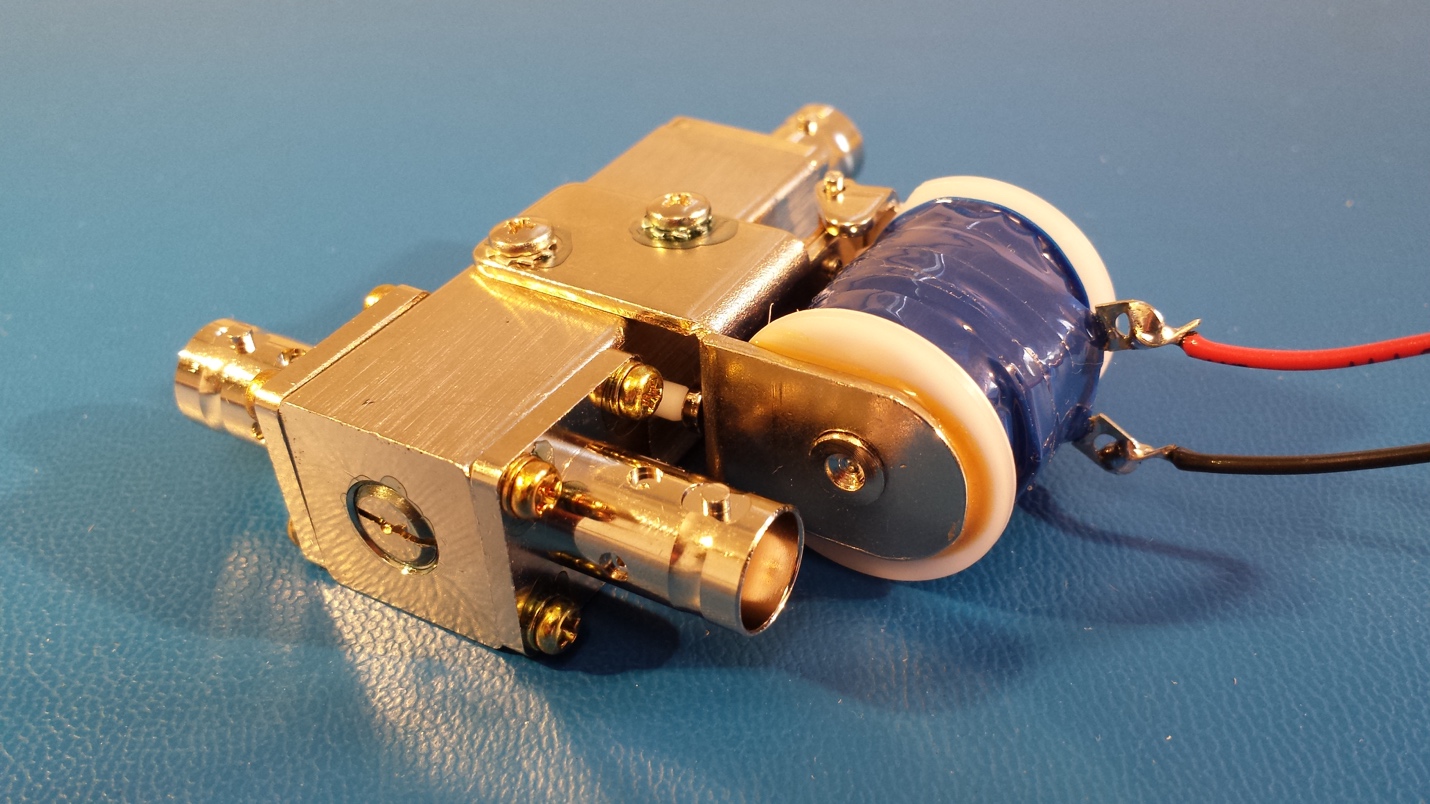


**Figure AM7:** Photograph of BNC Relay with wires soldered to coil terminals. (Reproduced, with permission, from Figure S3.4 of Kodandaramaiah et al. Nature Protocols 11, 634–654 (2016).

**STEP 3:** Solder 18” long red and black 24 G wires to the terminals of the BNC relay (Tohtsu CX-230). Repeat this step for all 4 channels.

**STEP 4:** Solder 18G, 12” long wires to the power circuit (**Fig. AM8**).


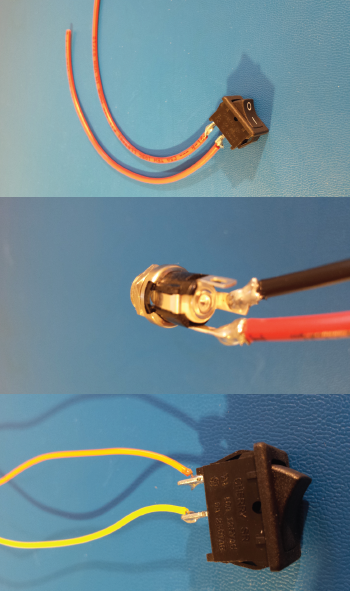


**Figure AM8:** Photograph illustrating preparation of the circuit elements (Adapted from Kodandaramaiah et al. 2016)

**STEP 5:** Prepare the potentiometers. Solder three 8” lengths each of orange, yellow, and black 24 gauge wire and wire them to the potentiometer leads as shown in **Figure AM9**. After soldering each potentiometer, test its function by rotating the control knob and checking resistance. Resistance between tabs 1 and 3 should be 50 K-Ohms. When turned fully clockwise, resistance between 1 and 2 should be 0 K-Ohms. Heat during soldering might cause the potentiometers to break. So, post testing is essential.


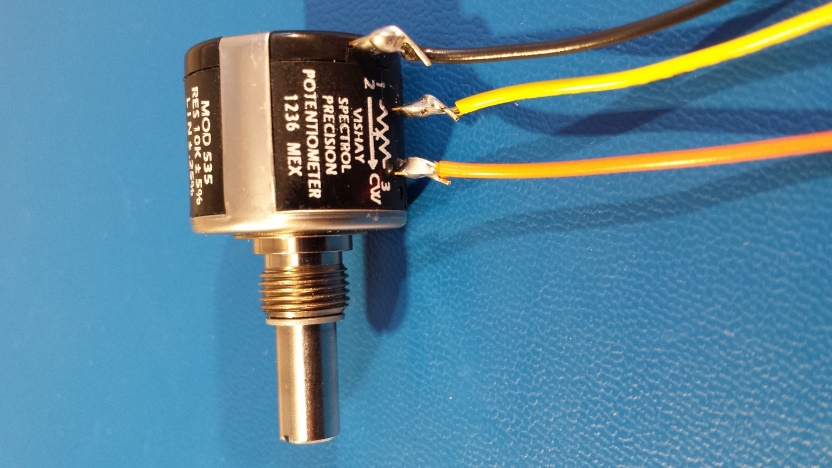


**Figure. AM9:** Preparation of the potentiometers. (Reproduced, with permission, from Figure S3.8 of Kodandaramaiah et al. Nature Protocols 11, 634–654 (2016).


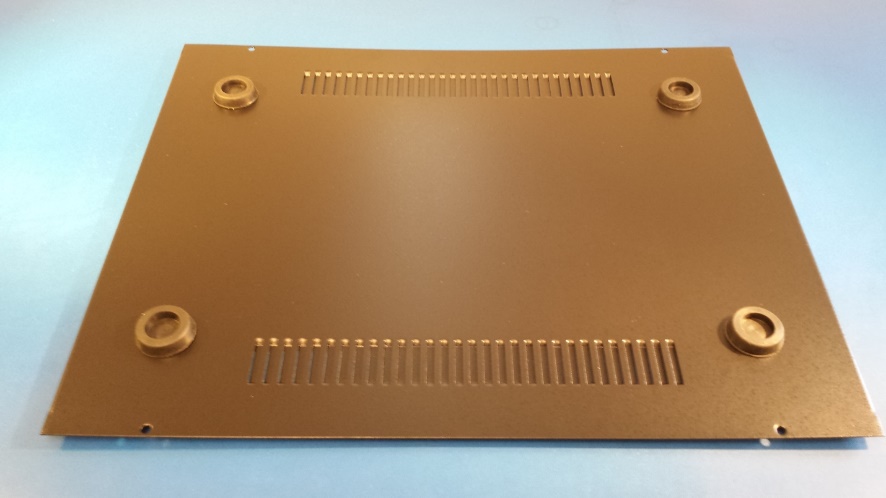


**Figure AM10:** Baseplate dampening. (Reproduced, with permission, from Figure S3.9 of Kodandaramaiah et al. Nature Protocols 11, 634–654 (2016).

**STEP 6:** Attached damper knobs to the base plates. This is done for both the control boxes.

**
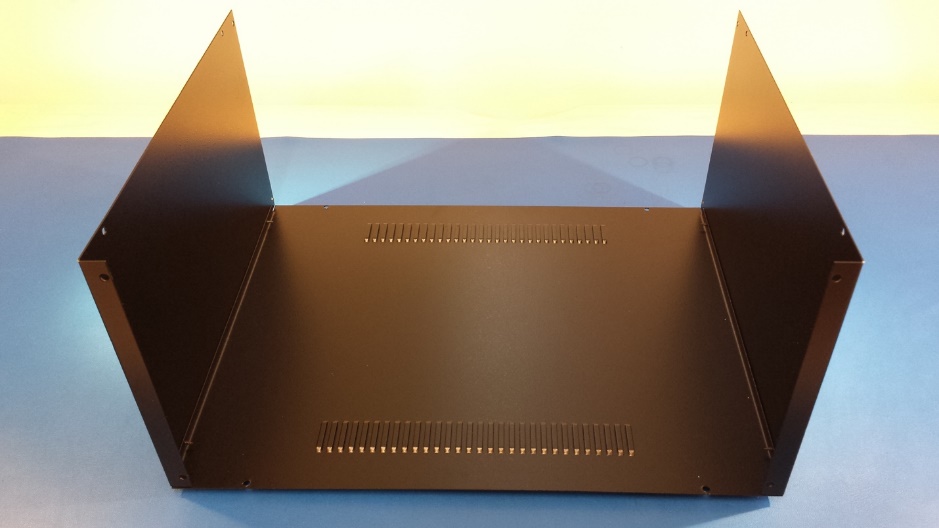
**

**Figure AM11:** Chassis pre-assembly (Reproduced, with permission, from Figure S3.10 of Kodandaramaiah et al. Nature Protocols 11, 634–654 (2016).

**STEP 7:** Assemble the chassis of each box by installing the side panels and as per manufacturer instructions. Do not assemble the front and back panels.

**STEP 8:** Add the nylon standoff to the base of the baseplate that have been laser cut. This step was not documented during the initial assembly of the multipatcher control hardware. Thus, this step is not illustrated here. Please see the assembly manual for the autopatcher control box for an equivalent illustration.

**STEP 9:** Attached the DAQ board to the signal switch box base panel suing fasteners provided by the manufacturer.


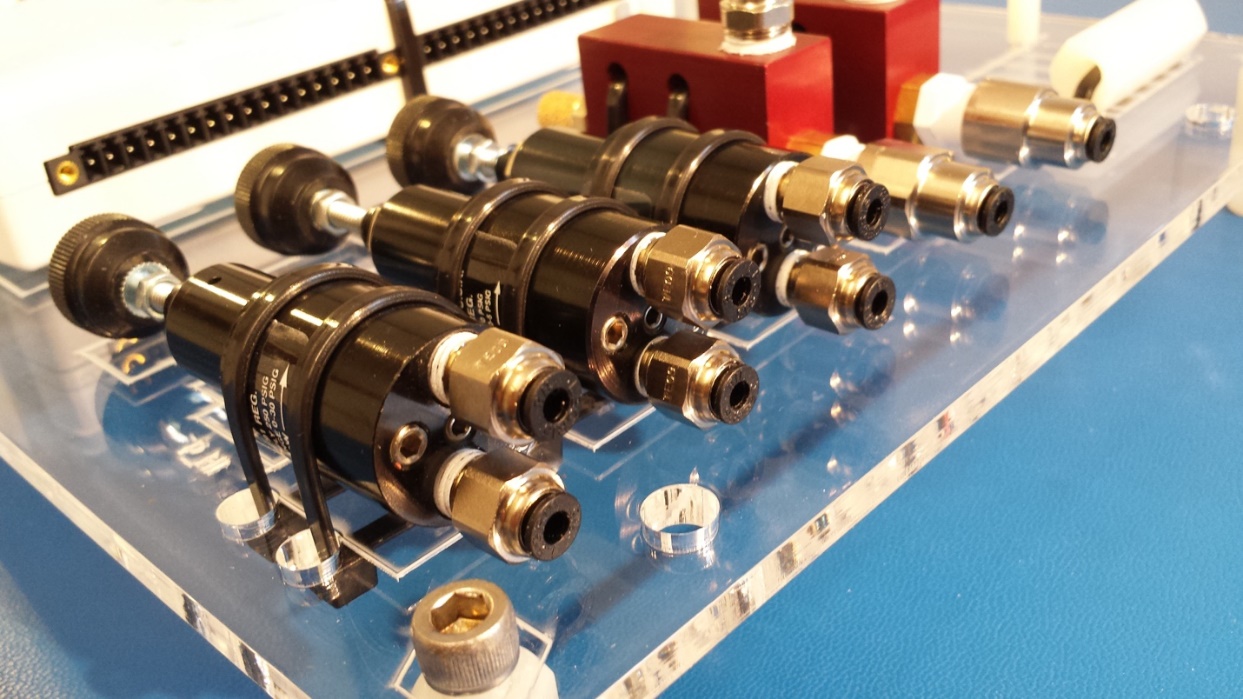


**Figure AM12:** Attaching manual pressure regulators to the base panel. (Adapted from Kodandaramaiah et al. 2016)

**Step 10:** Strap and manual and vacuum regulators to the base panel of the pressure control box (**Fig. AM12**).

**Step 11:** Assemble and fasten the electronic pressure regulators to the structural panel (**Fig. AM13**). This step was not documented during the initial assembly of the multipatcher control hardware. Thus, this step is not illustrated here. Please see the assembly manual for the autopatcher control box for an equivalent illustration.


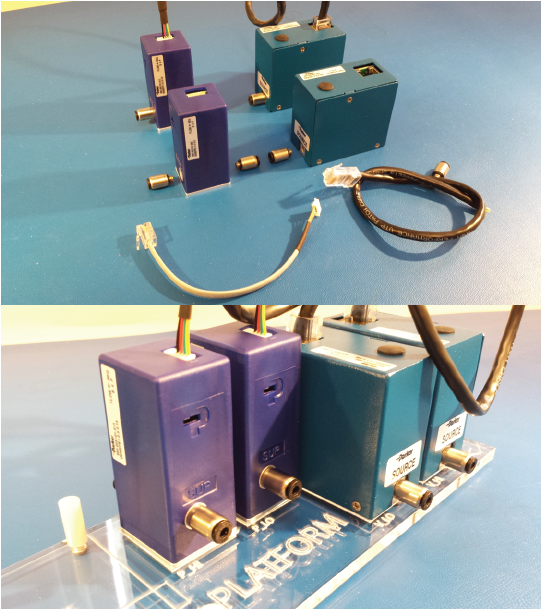


**Figure AM13:** Assembly and fastening of the electronic pressure controllers (Adapted from Kodandaramaiah et al. 2016).

**Step 12:** Mount the solenoid switch valves to the pressure switching valve boards and mount them on the support structure plate. Four boards with four valves each (**Fig. AM 14** left) and one board with two valves (**Fig. AM 14** right) need to be assembled.


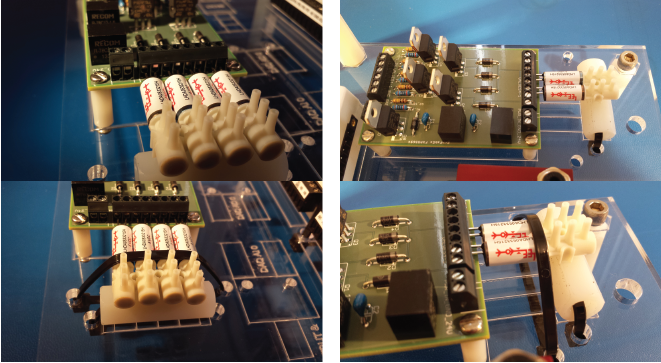


**Figure AM14:** Assembly of 4 valve and 2 valve control board. The board on the left has to be assembled and installed for each pipette channel that needs to be controlled by the multipatcher. (Photographs adapted from Kodandaramaiah et al. 2016.)


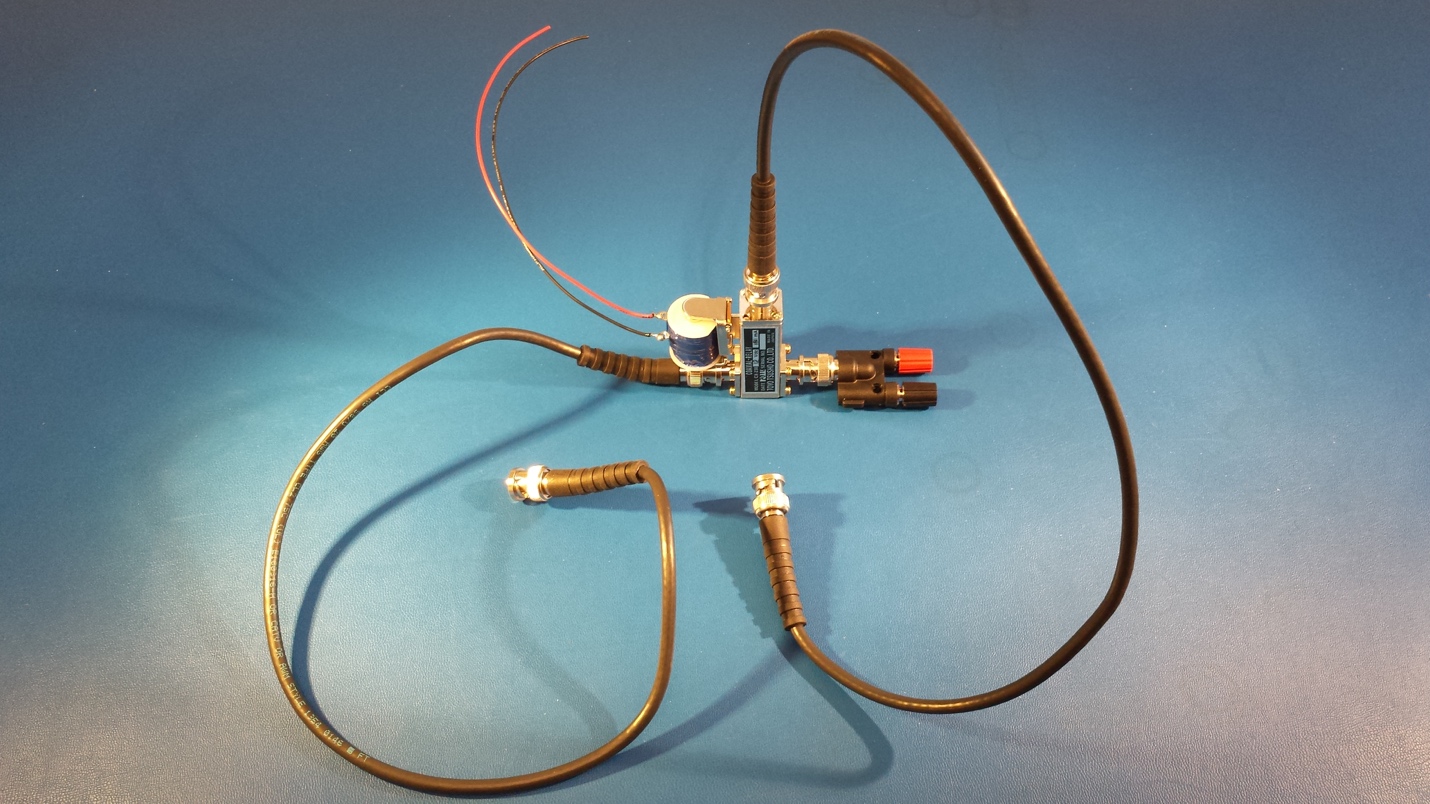


**Figure AM15:** Preparation of the BNC relay switch for mounting. (Reproduced, with permission, from Figure S3.27 of Kodandaramaiah et al. Nature Protocols 11, 634–654 (2016).

**Step 13:** Attached the BNC cables and female BNC binding post to the BNC relay switch. Repeat for all four channels. Secure it to the structural plate in the signal switch box using tie wraps (**Fig. AM15**).

**Step 14:** Secure the base plates to the rack mount chassis.

**Step 15:** Attach the USB adaptor plug, and he wall mount push to connect adaptors to the back panel of the multipatcher switch box.

**
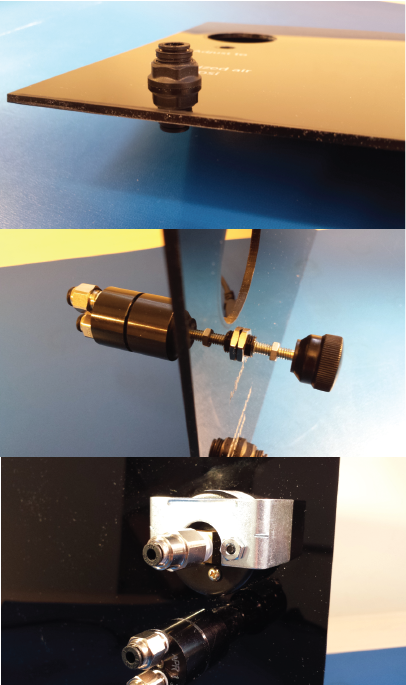
**

**Figure AM16:** Mounting fluidic components to back panel of the pressure control box. (Photographs adapted from Kodandaramaiah et al. 2016.)

**Step 16:** Attach the 0-60 psi panel mount pressure regulator, the ¼” through wall coupler to the hole marked 60 psi pressure and the wall mount manometer to the back panel of the pressure control box as shown, Repeat this step for the vacuum connections.


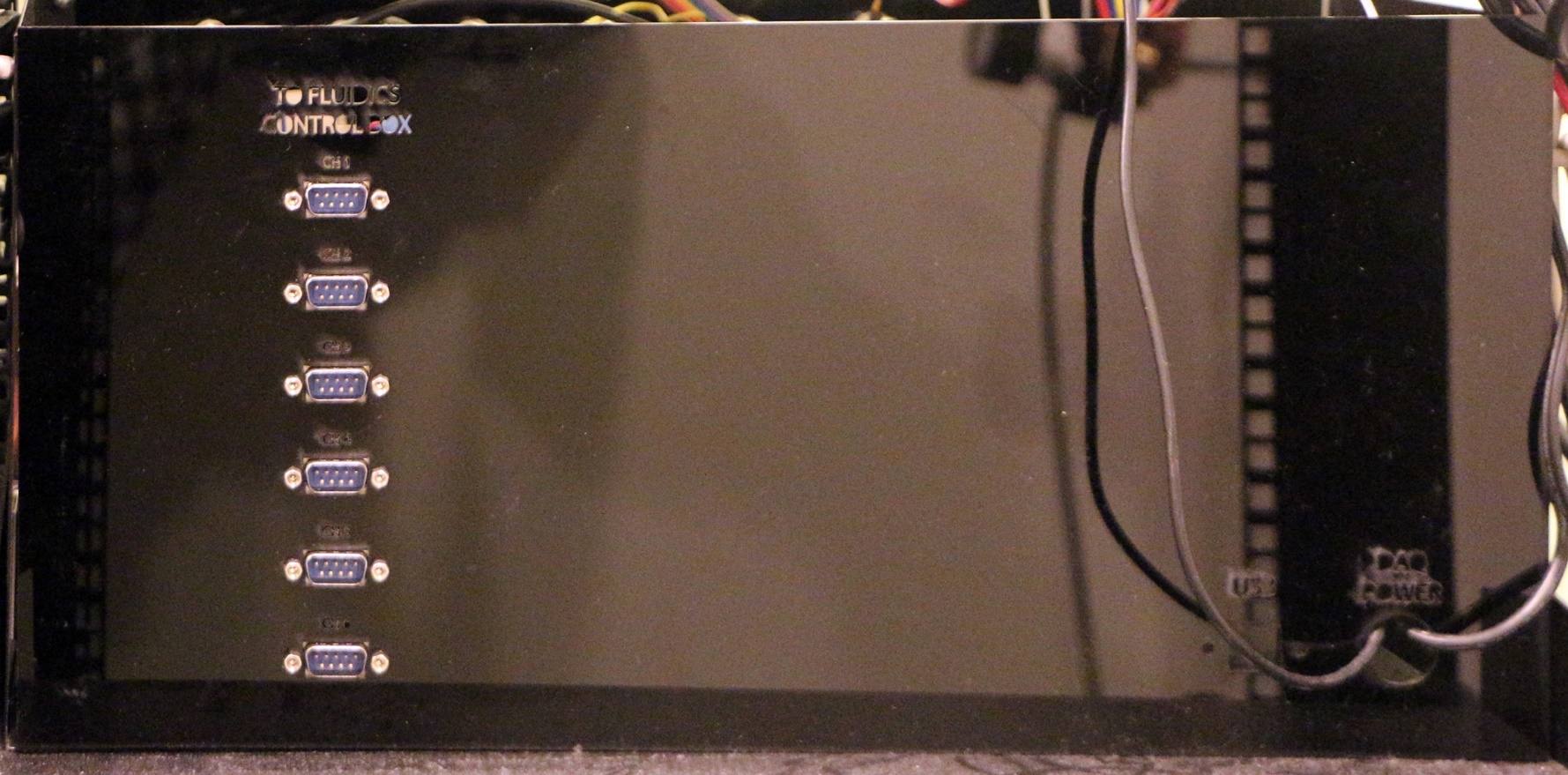


**Figure AM17:** Attaching DSUB connectors to the back panel of the pressure control box and signal control box.

**Step 17:** Mount the DSUB connectors to the back panels of the boxes.

**Step 18:** Attach the back panels to the chassis, ass illustrated in Step 36 of control box assembly manual of the autopatcher control box (Kodandaramaiah et al. 2016).

**Step 19:** Attach the wall mount 1/16” inch push to connect adaptor on the front panel of the pressure control box as described in Step 37 of the control box assembly manual of the autopatcher control box assembly manual (Kodandaramaiah et al. 2016).

**Step 20:** Attach the front panels to the two control boxes.

**Step 21:** Following the steps in the front panel assembly section in the autopatcher control box assembly manual (Section S3.3.4, autopatcher pressure control box assembly manual, Kodandaramaiah et al. 2016), attach the manometers and potentiometers to the front panel of the pressure control box. Similarly, attach the BNC wall mount plug adaptors to the front panel of the signal switch box. Finally, add the LED indicator, the power switch to the pressure control box front panel.

**Step 22:** Following the steps in the wiring section of the autopatcher control box assembly manual (Section S3.3.4, autopatcher pressure control box assembly manual, Kodandaramaiah et al. 2016), perform wiring to power the pressure control box.

**
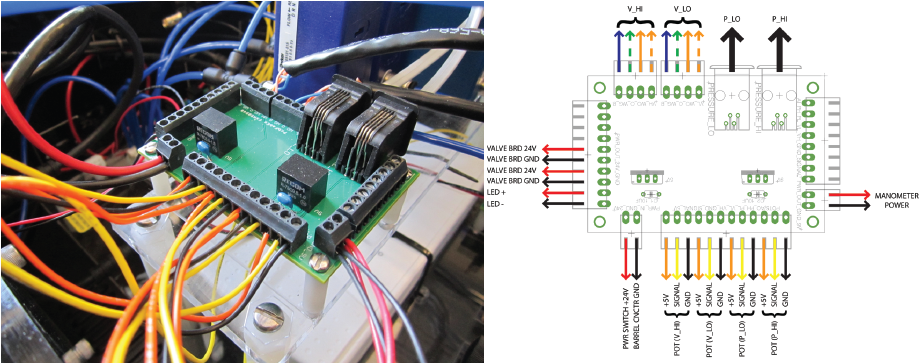
**

**Figure AM18:** Wiring the pressure regulator board (Adapted from Kodandaramaiah et al 2016)

**Step 23:** Wire the potentiometers, the pressure regulators, the wire to power the valve boards to the pressure regulator circuit as shown in **Fig. AM18**. Multiple pressure switching valve boards can be attached to the same power output. One board controls pressure output to one pipette channel.

**Step 24:** Upstream of the valve board, a single valve board is used to switch between high vacuum pressure and low vacuum pressure.

This is illustrated in **Fig. AM19**


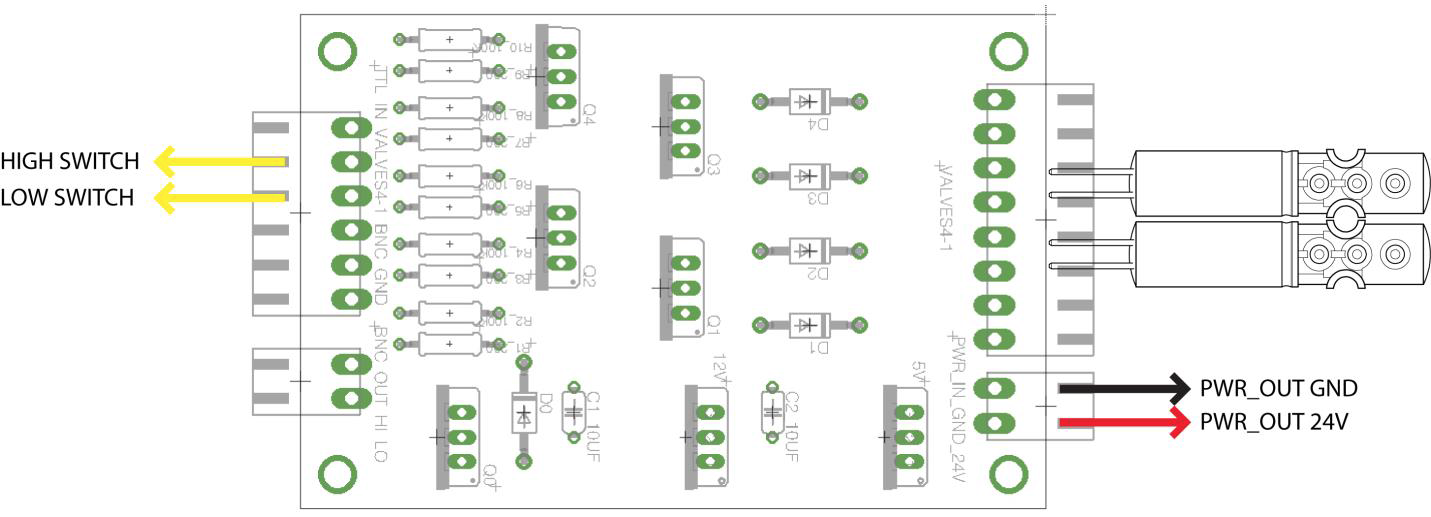


**Figure AM19:** Connections to the upstream vacuum switch valve (Reproduced, with permission, from Figure S3.58 of Kodandaramaiah et al. Nature Protocols 11, 634–654 (2016).

**Step 25:** Connect the analog voltage in and voltage out signals to the corresponding channels in the DAQ board. As shown in Fig. AM 20. Repeat this process for all the channels.


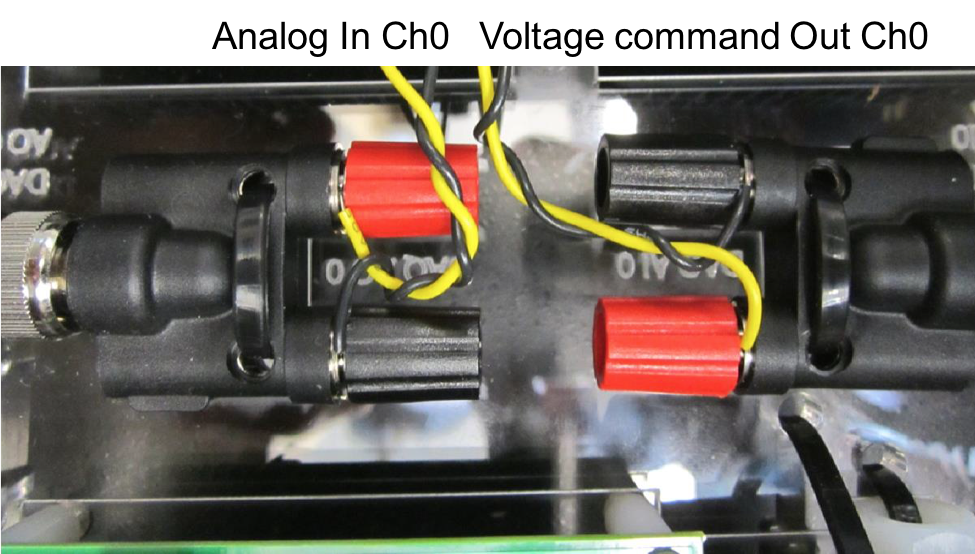


**Figure AM20:** Wiring from BNC splitter to each channel of the DAQ in the signal control box. (Adapted from Kodandaramaiah et al 2016)

**Step 26:** Wire the pipette valve board control TTL signals to the back panel’s serial cable. Keep note of the wiring. Ultimately, the signals through the serial port has to be connected to each valve board using the schematic shown in Fig. AM 21.


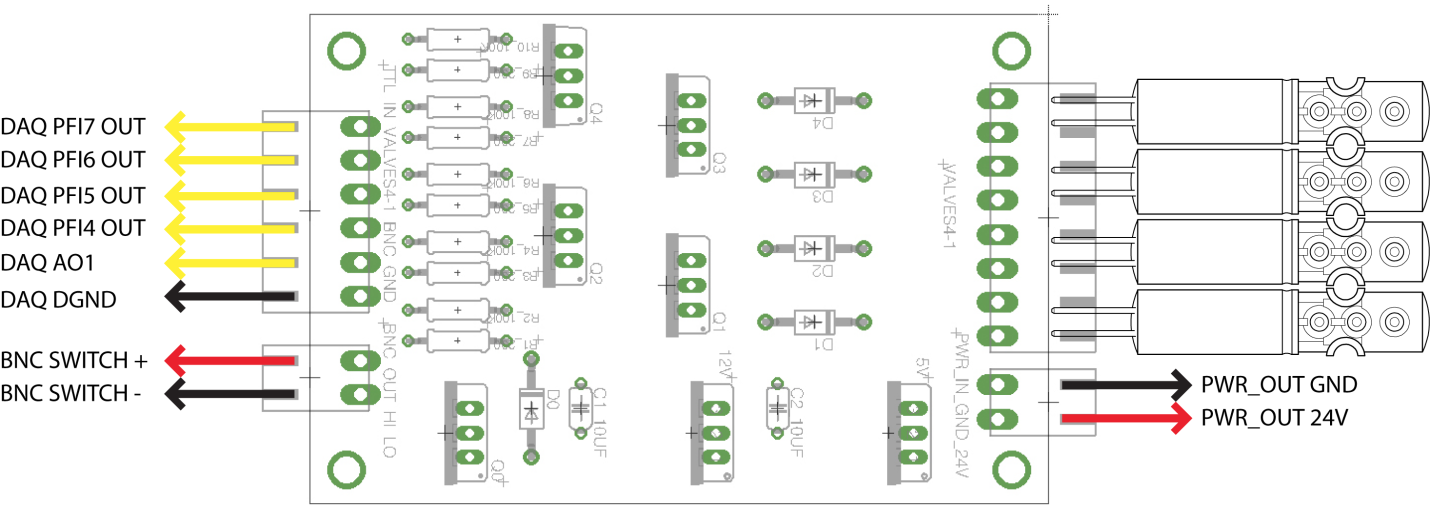


**Figure AM21:** Wiring from DAQ board to one pressure valve board. (Adapted from Kodandaramaiah et al 2016)

**Step 25:** Fluidic connections need to be made next to ensure pressures are properly regulated during multipatching. These instructions are identical to the instructions for performing these tasks for the single channel autopatcher. See ‘Section 3.3.6 Internal plumbing’ in Kodandaramaiah et al. 2016. Follow those instruction to first plug in the upstream vacuum control board, followed by fluidic connections to each pressure valve board. For reference, the fluidic connection scheme are illustrated in **Fig. S3.67 and S3.68** in Kodandaramaiah et al. 2016.

**Step 26:** This is followed by fluidic circuit calibration and signal circuit testing, which are again comprehensively detailed in the autopatcher user manual. In the case of the multipatcher, serial DB 9 cables connect the signal switch box to the pressure control apparatus. Further, once the pressures states have been calibrated, each channel needs to be independently tested for proper pressure switching and signal interfacing.

The hardware is now ready for multipatching.
