# Supplementary material for: Multi-neuron intracellular recording in vivo via interacting autopatching robots
Source: eLife. 2018 Jan 3;7:e24656. doi: 10.7554/eLife.24656 (PMC5812718; doi:10.7554/eLife.24656)
Supplement: Supplementary file 4. — The Supplementary file 4 describes the installation and use of the multipatcher control software under the LabView system-design platform. [file elife-24656-supp4.docx]

**Supplementary file 4.**

**Multipatcher Software Setup and Operation.**

The multipatcher software’s graphic user interface (GUI) is organized into five tab. There are ‘controls’ that allow the user to specify various parameters into the program for multipatching, and several displays that display statuses and data during multipatcher operation. Below is a brief overview of the GUI followed by instructions for setting up and running the multipatcher.


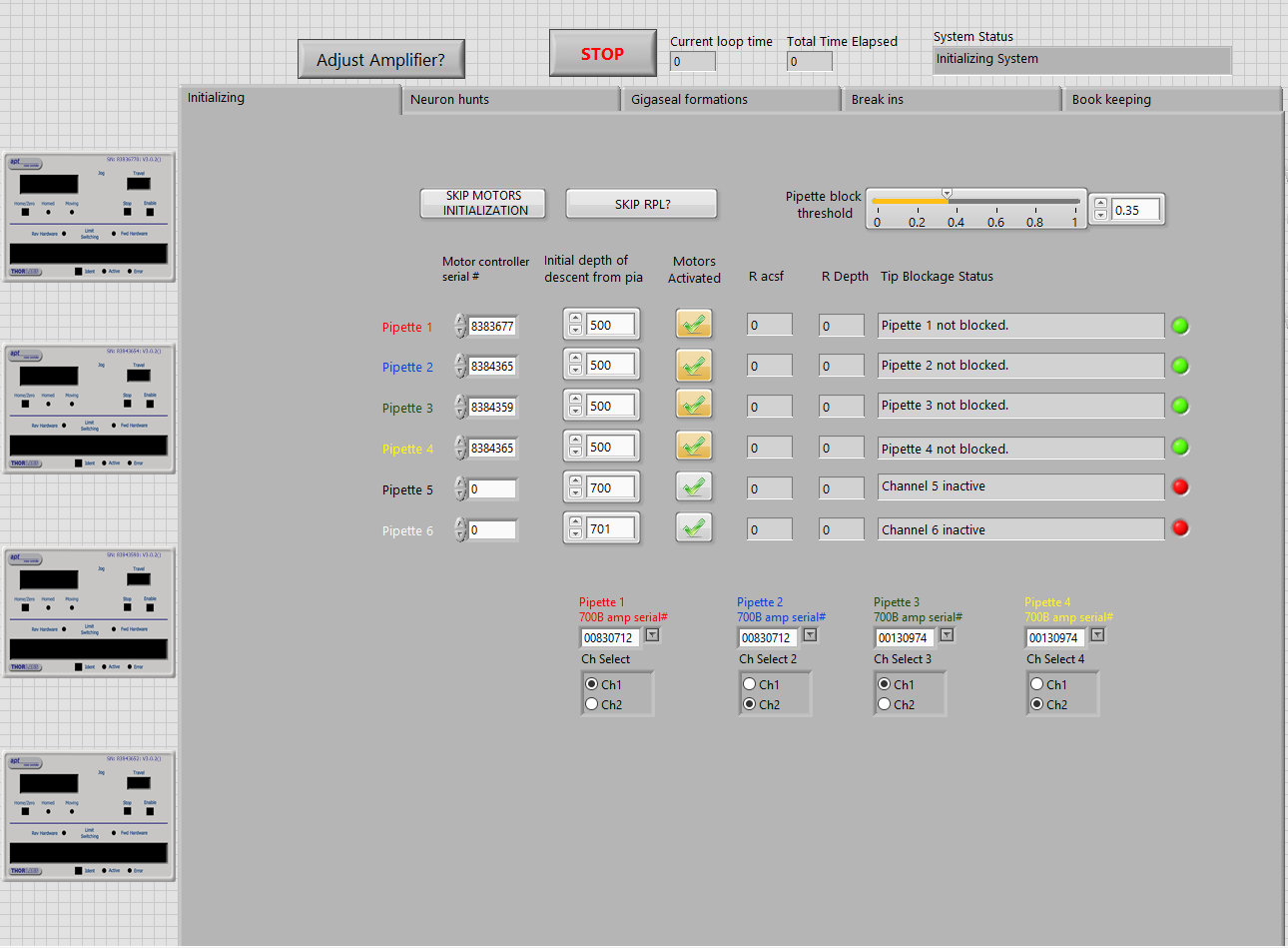


**Figure SM1:** Front panel of the multipatcher software graphic user interface (GUI) at the beginning of multipatcher operation.

**Initializing Tab:**

**‘Adjust amplifier?’ –** If checked will automatically communication with Multiclamp Commander software, and correct for any pipette offsets and pipette capacitances before beginning multipatching.

**‘STOP’** – Aborts the multipatching trial at any point.

**‘Current loop time’** - Displays the time taken for the program to complete a loop. Typically loop time varies based on number of times the pipette resistance is averaged during neuron hunting.

**‘Total time elapsed’ –** Indicates the total time since the beginning of the multipatcher trial. This includes the time taken to install the pipettes.

**‘System Status’** – Displays the instantaneous status of the multipatcher operations. These are: ‘Initializing systems’, ‘Checking pipette resistances in saline’, ‘Lowering active pipettes into the brain’, ’Neuron hunting’, ‘Gigasealing’, and ‘Breaking in’.

**‘Skip Motors Initialization’** – The initialization of the motors functions allows LabVIEW to communicate with the Thorlabs’s control program via .NET protocols. This typically takes 15-20 seconds, and needs to be run only in the first instance the program is run in LabVIEW. Checking the Skip Motors Initialization button, saves that time in subsequent runs of the program.

**‘Skip RPL?’** - This control allows the user to manually insert pipettes into the brain for neuron hunting, in which case the automatic execution of insertion of pipettes into the brain is skipped.

**‘Pipette block threshold’** – Some fraction of the pipettes get blocked or clogged when inserted into the brain in the initial descent to depth. This is typically accompanied by an increase in pipette resistance. This control allows the user to set the threshold for increase in pipette resistance. We have used a threshold of 0.35 M-Ohms, through this study and previous studies (Kodandaramaiah et al. 2012, Kodandaramaiah et al. 2017).

An array of controls and indicators specific each pipette is displayed underneath these controls.

**‘Initial depth of descent from pia’** - The user can independently set the depths to which each pipette is lowered in the initial step. The pipettes hunt for neurons starting from this depth.

**‘Motors activated’** – The user can choose the active channels used in a particular trial.

**‘R acsf’** – The multipatcher displays the resistance measured in the ACSF solution outside the brain surface of the active pipettes.

**‘R depth’** – The multipatcher displays the resistance measured inside the brain after the initial descent to depth.

**‘Tip blockage status’** – The multipatcher displays whether a pipette is okay, blocked or is not active.

**‘Pipette x amplifier serial number’** - At the bottom of the tab, we have controls that allow the user to select the serial number of the Axon Multiclamp 700b amplifier and the channel corresponding to a particular pipette.

**‘Motor controller serial #’ –** Allows the user to input the serial numbers of the controllers driving the linear motors in labview so they can be programmatically controlled during multipatching.


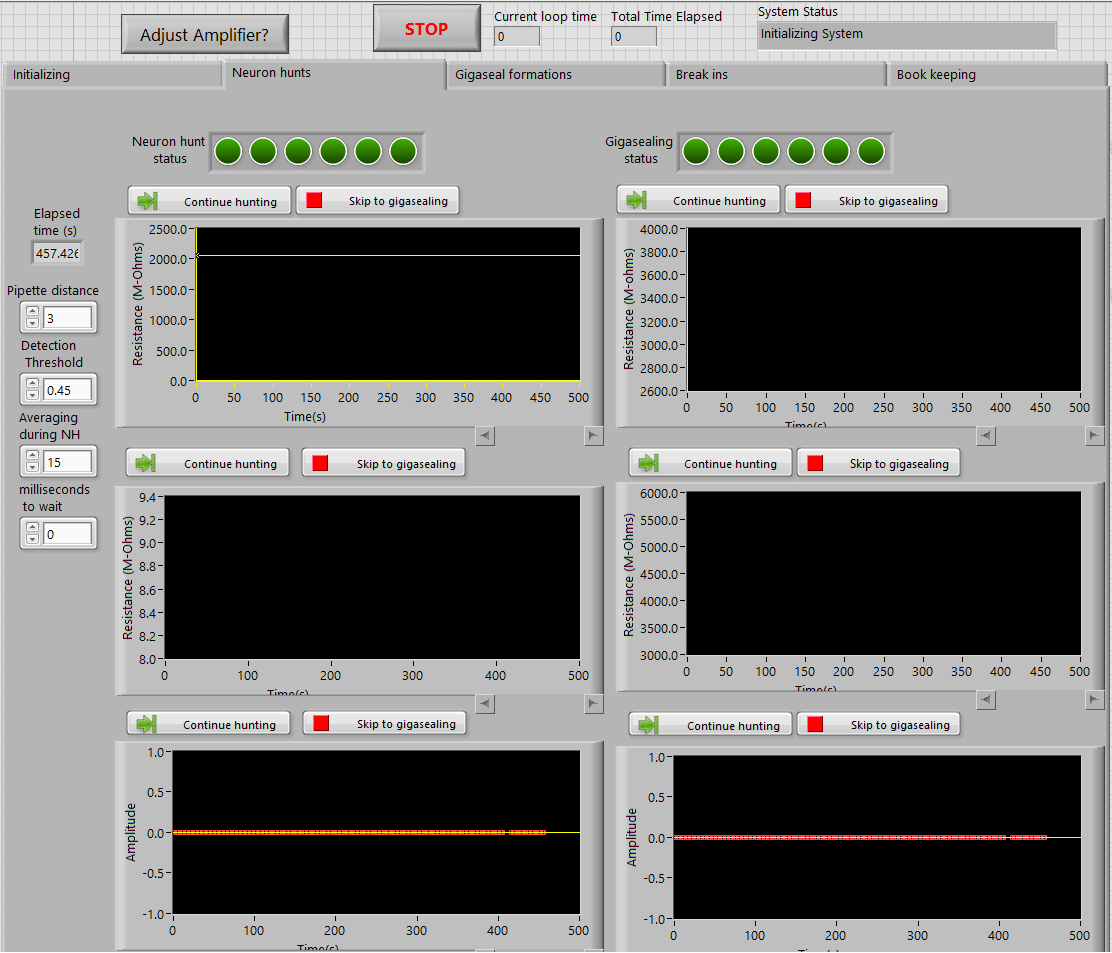


**Figure SM2:** Front panel of the multipatcher software GUI displayed during neuron hunting.

**Neuron Hunting Tab:**

**Figure SM2** displays the multipatcher software GUI displayed during the neuron hunting stage of multipatching. Each pipette has a corresponding graph that displays the measured resistance as a function of time.

**‘Continue hunting’** – This control allows the user to override the multipatcher algorithm’s neuron detection algorithm and force the pipette to continue searching for neurons.

**
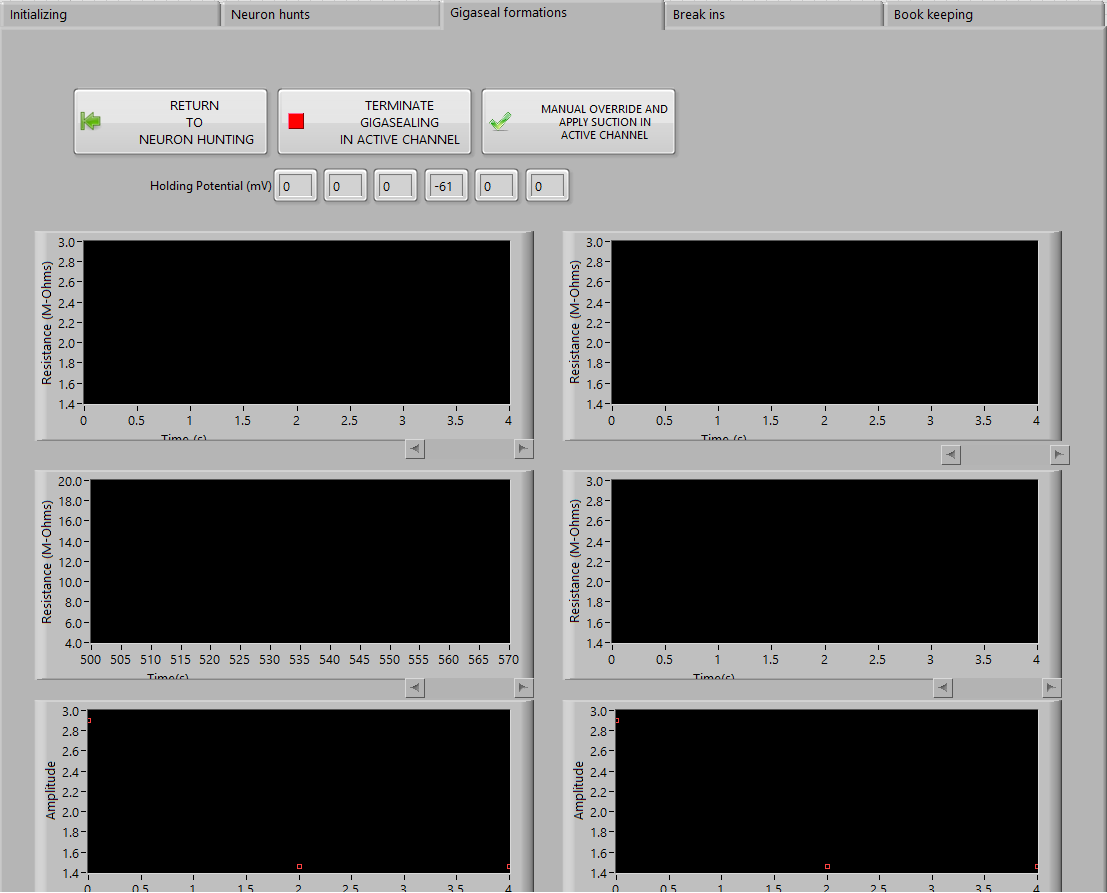
**

**Figure SM3:** Front panel of the multipatcher software GUI displayed during gigasealing

**‘Switch to gigasealing’** - this control allows the user to override the lack of detection of neurons in a particular channel by the algorithm and attempt instantaneous gigasealing.

**‘Elapsed time’** - Displays the time elapsed in seconds since beginning of neuron hunting.

**‘Pipette distance’** – Allows the user to set the distance moved by the pipette in each step before resistance measurement. This was typically set at 3 micrometers.

**‘Detection threshold’** – This is the threshold that resistances over two steps needed to monotonically increase before the algorithm positively identified contact with a cell. It was set at 0.25-0.45 (M-Ohms)

**‘Averaging during NH’** – Specifies the number of resistance measurements taken at the end of each step during neuron hunting. This was set at 15 measurements.

**‘milliseconds to wait’** – Was a time delay control we used during initial debugging to delay the stepping forward during neuron hunting. This was always set at zero during the experiments.

**‘Neuron Hunt status’** – Indicate whether a particular channel (1-6, left to right) had encountered a neuron.

**‘Gigasealing Status’** – Indicate whether a particular channel (1-6, left to right) had completed the gigasealing operation.

**Gigasealing Tab:**

The ‘Gigasealing’ tab (**Fig. SM3**) has controls and indicators related to the gigasealing process and is displayed whenever gigasealing is attempted in any one of the pipettes. Each pipette has an indicator where pipette resistances are measured every 0.5s during the gigasealing process.

There are also indicators that display the instantaneous holding potential applied to each pipette. This is set at zero initially and is stepped down to -35mV after 25 seconds when suction application is removed and ramped down to -70 mV. There are three manual override controls that present in this tab.

**‘Return to neuron hunting’** – Allows the user to override the gigasealing operation and return back to neuron hunting if he/she thinks the pipette picked up a false positive. While the algorithm automatically does this at the end of ten seconds if the pipette resistance does not stay elevated, this allows the user to save a few seconds which might be critical especially when patching in awake animals.

**‘Terminate gigasealing in active channel’** - This control can be used by the user to terminate the gigasealing process prior to the 60s cut off to save time if (i) gigasealing has already resulted in good seal or (ii) it appears that the pipette is unlikely to seal onto the cell. This control was used at point (iv) in the trial illustrated in Fig. 4 of the main paper. This control is again critical for saving time particularly when patching in awake animals. Using this control results in the pipette pressure set to atmospheric pressure and application of -70 mV holding potential, after which the neuron hunting is resumed. If this option is used in the last active pipette, the program proceeds to the breaking in stage.

**‘Manual override and apply suction in active channel’ –** The multipatcher applies suction pressure to the pipette during 15-25 s of the gigasealing operation. This control allows the user to override the pressure modulation routine as shown in Fig 4a and apply suction anytime during the gigasealing operation.

**Breaking-in Tab:**

The final tab is the breaking in tab that is illustrated in **Figure SM4**. The book keeing tab consists of various vestigial displays that were used when coding the algorithm and are not required for multipatcher operation.


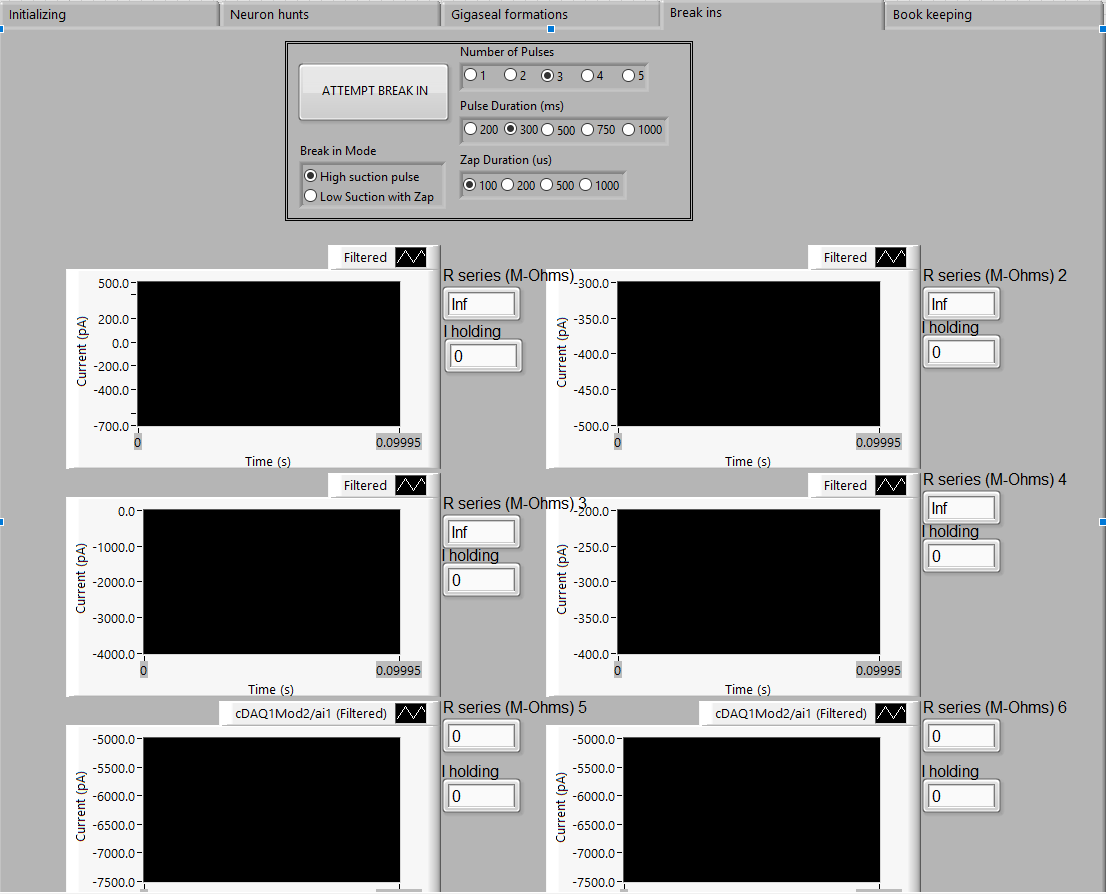


**Figure SM4:** The breaking in tab in the multipatcher software GUI.

The main control elements here are the ‘Attempt break in’ button and the associated breaking in parameter controls. The program allows the user to choose between two modes of breaking in - pure high suction pulses and low suction pulses accompanied by simultaneous voltage ‘zap’ administered by the amplifier headstage. If using the high suction pulse mode, the user has the options to vary the number of pulses and the pulse duration. If using the low suction pulse with zap mode, the multipatcher applies low suction pressure with user having control over the zap duration.

Each pipette has a corresponding ‘whole cell currents’ graph indicator that display the currents measured by the amplifier in response to a 50 Hz square wave voltage pulse. This allows the user to confirm whether breaking in was successful. Adjacent numerical displays indicate the measured series resistance as well as the holding current being injected to voltage clamp the cell at -70 mV.

**Multipatcher Software Configuration:**

1. Download and install the latest DAQmx driver software from NI.com

2. Obtain a license and install the latest version of LabVIEW. It has to be version 2011 or later.

3. Install Thorlabs APT software that controls the linear motors

4. Open the Multipatcher.llb file.

5. Open Multipatcher_Ver0.77_Thorlabsmotors.vi. You should see the screen illustrated in **Fig. SM1** in LabVIEW.

6. Note down the serial numbers of motor controller attached to each of the motors, and enter them at the corresponding ‘Motor controller serial #’ controls. This allows the software to communicate with the motor controllers. After an initialization period (~10 seconds) the motor controller panels on the left will display the positions of the motors.

8. Pressing Ctrl + E switches the LabVIEW window to the block diagram. Ensure the DAQ name at the beginning of the block diagram (LabVIEW programs run visually left to right), corresponds to the automatically generated device address that is shown in NI-MAX software.

9. This should get the multipatcher configured for multipatching.

**Running the Multipatcher:**

10. Once configured as detailed in Step 9, the default values for the controls in the multipatcher software should allow smooth operation. However, the user may need to change the pipette descent depths in the opening tab.

11. Run the multipatcher software by clicking the right arrow button in the LabVIEW window. This will reset the valves to deliver high positive pressure to the pipette holders. A pop-up dialog box will appear instructing the user to install the pipettes and prepare the robot for patching.

12. Fill and install the pipettes in the pipette holder. Using the stereomicroscope for visualization, lower the pipettes to the corresponding craniotomies until they just make contact with the brain. Once all pipettes are positioned, click ok in the pop-up dialog box. The multipatcher will do the rest of the patching until the breaking in step.

13. Chose the channel you want to break-in into and select appropriate pressure setting. Clicking attempt break-in button will results in a pop-up dialog box that guides the user through the breaking in procedure. Options are given to choose the channels to break-in, the break in pressure pulse pressure setting, duration and option of including a co-incident zap during application of pressure. This process may have to be repeated 1-5 times to achieve successful break in.

14. Immediately upon pressure pulse application, a second pop-up dialog box appears which instructs the user to choose to either break in again, or exit the program to record. Upon exiting, the amplifiers are automatically set to I = 0 mode before changing over to Clampex and Multiclamp Commander software for recording.
