# Supplementary material for: Multi-neuron intracellular recording in vivo via interacting autopatching robots
Source: eLife. 2018 Jan 3;7:e24656. doi: 10.7554/eLife.24656 (PMC5812718; doi:10.7554/eLife.24656)
Supplement: Transparent reporting form [file elife-24656-transrepform.docx]

***eLife’s* transparent reporting form**

We encourage authors to provide detailed information *within their submission* to facilitate the interpretation and replication of experiments. If you have any questions, please contact us: [editorial@elifesciences.org](mailto:editorial@elifesciences.org).

**Sample-size estimation**

- You should state whether an appropriate sample size was computed when the study was being designed
- You should state the statistical method of sample size computation and any required assumptions
- If no explicit power analysis was used, you should describe how you decided what sample (replicate) size (number) to use

Please outline where this information can be found within the submission (e.g., page numbers or figure legends), or explain why this information doesn’t apply to your submission:

We did not compute the sample size because the experiments were performed to demonstrate the efficacy of the methodology presented, and not to make statistical claims about a biological phenomena or mechanism.

**Replicates**

- You should report how often each experiment was performed
- You should include a definition of biological versus technical replication
- The data obtained should be provided and sufficient information should be provided to indicate the number of independent biological and/or technical replicates
- If you encountered any outliers, you should describe how these were handled
- Criteria for exclusion/inclusion of data should be clearly stated
- High-throughput sequence data should be uploaded before submission, with a private link for reviewers provided (these are available from both GEO and ArrayExpress)

Please outline where this information can be found within the submission (e.g., page numbers or figure legends), or explain why this information doesn’t apply to your submission:

In this study, the main biological unit of interest is the neuron. Therefore, all replicates are biological replicates because of the impossibility of patching more than once in the same neuron. However, we indicate throughout the text whenever we performed more than one patching trial in the same animal.

To assess performance of the methodology, we also discuss individual mice as biological unit of interest, and two non-biological units: number of pipettes and number of trials. It is indicated throughout the text whenever this is the case.

**Statistical reporting**

- Statistical analysis methods should be described and justified
- Raw data should be presented in figures whenever informative to do so (typically when N per group is less than 10)
- For each experiment, you should identify the statistical tests used, exact values of N, definitions of center, methods of multiple test correction, and dispersion and precision measures (e.g., mean, median, SD, SEM, confidence intervals; and, for the major substantive results, a measure of effect size (e.g., Pearson's r, Cohen's d)
- Report exact p-values wherever possible alongside the summary statistics and 95% confidence intervals. These should be reported for all key questions and not only when the p-value is less than 0.05.

In this study, only one test of significance was performed (page 12). The methodology used for statistical analyses is reported there, including the statistical test used and exact p-value. The choice of the test was based on the robustness of non-parametric hypothesis testing to non-normality of the data.

Exact values of N and measures of center and dispersion are defined and presented throughout the text. Because the main focus of this paper is to present a novel methodology, we are not reporting effect size because we are not making claims about treatment effects.

Please outline where this information can be found within the submission (e.g., page numbers or figure legends), or explain why this information doesn’t apply to your submission:

(For large datasets, or papers with a very large number of statistical tests, you may upload a single table file with tests, Ns, etc., with reference to page numbers in the manuscript.)

**Additional data files (“source data”)**

- We encourage you to upload relevant additional data files, such as numerical data that are represented as a graph in a figure, or as a summary table
- Where provided, these should be in the most useful format, and they can be uploaded as “Source data” files linked to a main figure or table
- Include model definition files including the full list of parameters used
- Include code used for data analysis (e.g., R, MatLab)
- Avoid stating that data files are “available upon request”

Please indicate the figures or tables for which source data files have been provided:

We have uploaded the raw data for the data shown in figures 4, 5 and 6.

We used the Clampfit software, which is publicly available, for visualization of current clamp recordings.

We used MATLAB code for motion analysis, available at <https://github.com/fjflores/VideoMotionEstimation>.

The multipatcher software runs in the LabView platform, and we are making all the necessary code available with the manuscript.

Figures 4, 5, and 6.
